# China Medicinal Plants of the *Ampelopsis grossedentata*—A Review of Their Botanical Characteristics, Use, Phytochemistry, Active Pharmacological Components, and Toxicology

**DOI:** 10.3390/molecules28207145

**Published:** 2023-10-18

**Authors:** Rong-Rong Wu, Xiang Li, Yu-Hang Cao, Xiong Peng, Gao-Feng Liu, Zi-Kui Liu, Zi Yang, Zhao-Ying Liu, Yong Wu

**Affiliations:** 1 College of Veterinary Medicine, Hunan Agricultural University, Changsha 410128, China; 18390873915@163.com (R.-R.W.); 18374954319@163.com (Y.-H.C.); 113548586471@163.com (X.P.); lzk7035@sina.com (Z.-K.L.); 2 Hunan Canzoho Biological Technology Co., Ltd., Liuyang 410329, China; plantgene@163.com (X.L.); canzoho2022@126.com (G.-F.L.); 3 Academician Workstation, Changsha Medical University, Changsha 410219, China; yangziycy@163.com

**Keywords:** *Ampelopsis grossedentata*, China medicinal plants, phytochemistry

## Abstract

*Ampelopsis grossedentata* (AG) is mainly distributed in Chinese provinces and areas south of the Yangtze River Basin. It is mostly concentrated or scattered in mountainous bushes or woods with high humidity. Approximately 57 chemical components of AG have been identified, including flavonoids, phenols, steroids and terpenoids, volatile components, and other chemical components. In vitro studies have shown that the flavone of AG has therapeutic properties such as anti-bacteria, anti-inflammation, anti-oxidation, enhancing immunity, regulating glucose and lipid metabolism, being hepatoprotective, and being anti-tumor with no toxicity. Through searching and combing the related literature, this paper comprehensively and systematically summarizes the research progress of AG, including morphology, traditional and modern uses, chemical composition and structure, and pharmacological and toxicological effects, with a view to providing references for AG-related research.

## 1. Botanical Description

There are approximately 30 species of vitaceae and ampelopsis plants in the world, and approximately 17 species and mutations are distributed in China, most of which are endemic to China and mainly distributed in hillside shrubs and forests in the southwest, south, and northeast of China [1]. *Ampelopsis grossedentata* (AG) is a genus of ampelopsis in the family Rutaceae of the angiosperm family Magnoliaceae [2]. The roots of the whole plant are slender, fibrous, and partially curved. They have climbing, branched stems with longitudinal ribs on the surface. Moreover, they are glabrous, and the nodes are dilated. The tendrils are bifurcated and spaced two internodes apart on opposite sides of the leaves [3]. The leaves are bipinnate, petioles are 1.5–3.0 cm long, stipules are caducous, the upper leaves of the branches are almost sessile, apical leaflets have petioles, lateral leaflets are sessile, and both sides of the leaves are glabrous. The cymes arise from leaf axils or branch apices opposite the leaves. The calyx is discoid and 2.2 mm in diameter. They have five oblong-shaped petals and five stamens, and the flower disc is shallowly cup-shaped. The fruit is a berry, which is nearly spherical and purple-black when mature, with a diameter of 3–6 mm. Their flowering period is from June to September, and the fruiting period is from July to November [4].

## 2. Use

### 2.1. Traditional Uses

AG has a long history of use in China as an ancient medicinal and food homologous plant. The Chinese folk drink AG dates back 600 years to the period of “Divine Farmer“ testing a hundred varieties of herbs.” People of the Zhuang and Yao nationalities were the first to utilize it [5]. Afterward, it was extensively used in the Tujia, Lahu, Dong, Jino, and Hakka nationalities. AG was first recorded in the Classic of Tea [6], which has the functions of clearing heat and detoxifying, relieving cough and phlegm, promoting blood circulation and dredging collaterals, protecting the liver, and dispelling rheumatism [7]. Since then, ancient texts such as “Ying Shan Zheng Yao” [8] and “Cao Mu Bian Fang” [9] have used this name to record AG and its effects. The usage of AG was recorded in the “Chinese Materia Medica,” mostly for internal use, using 15–30 g of AG in decoction or tea making. The nickname and traditional use of rattan tea vary in each region, and the specific content is presented in Table 1.

### 2.2. Modern Uses

Common products on the market include various AG drinks and “Hua An Baimaohou” boxed products refined from the tender stems and leaves of AG [21]. Luo Anling [22] combined AG, *Lobed Kudzuvine* root, and corn oligopeptide into a compound formula and conducted an intervention test on the drunk mouse model. The results showed that the compound formula down-regulated the expression levels of NF-κB and TNF-α, thereby improving liver tissue damage and reducing the extent of liver cell damage caused by alcohol. Zhang et al. [23] formulated a creamy yellow soymilk-AG compound drink composite drink with a 5:1 ratio of soymilk and AG juice volume with 0.03% sucralose and 0.2% carboxymethyl cellulose as a stabilizer. The formulation had a bean fragrance, a slight AG fragrance, and high nutritional and therapeutic value. The AG and ginseng capsules prepared from AG, *Codonopsis ginseng*, *Lycium barbarum*, and starch have the effects of lowering blood glucose, blood lipids, and blood pressure, enhancing immunity, and delaying aging [24]. The compound tea bag made of AG, Qingqian willow, and mulberry leaves lowers blood glucose, and the compound tea bag is more convenient to brew. The liquid when tea bags are brewed in boiling water is transparent, has the unique aroma of AG, and is rich in total flavonoids and polysaccharides, which have good market prospects [25]. In the study of Zheng et al. [26], AG was extracted with water at 80 °C for 30 min. Sucrose, salt, and citric acid were added at 7.40%, 0.09%, and 0.04%, respectively, and a sucrose-type AG beverage was prepared. The formula can be blended into a xylitol-type AG beverage by adding 9.08%, 0.08%, and 0.05% of xylitol, salt, and citric acid, respectively. 

## 3. Phytochemistry

**Figure 1:**
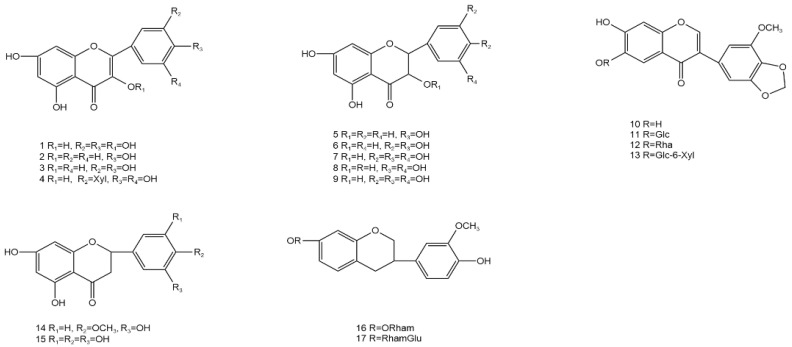
Structural formula of flavonoids in AG (**1**–**17**).

**Figure 2:**
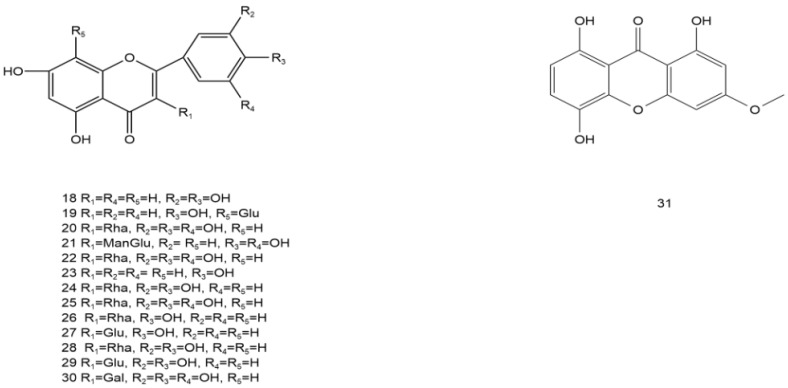
Structural formula of flavonoids in AG (**18**–**31**).

**Figure 3:**
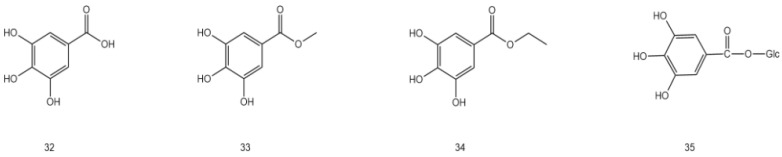
Structural formula of phenolic compounds in AG.

**Figure 4:**
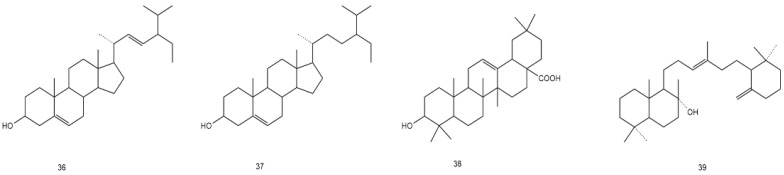
Structural formula of steroids and terpenoids in AG.

**Figure 5:**
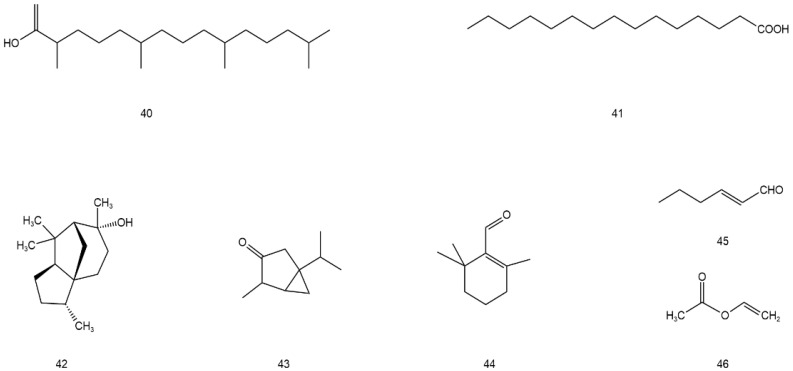
Structural formula of volatile components and other compounds in AG (**40**–**46**).

**Figure 6:**
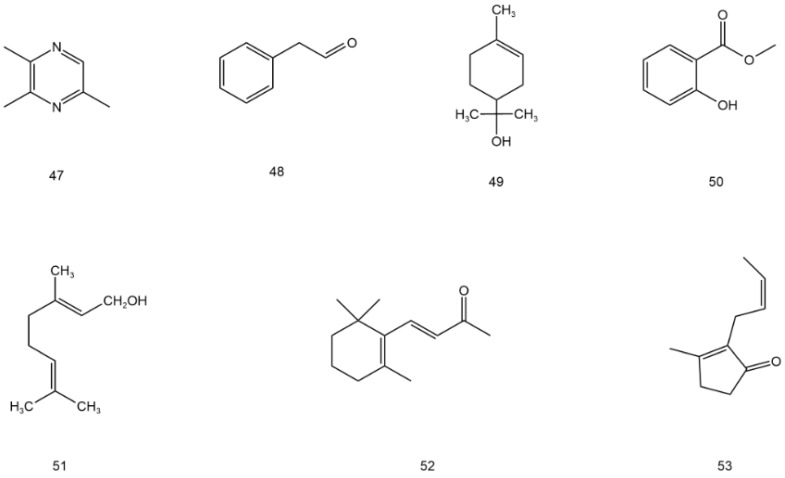
Structural formula of volatile components and other compounds in AG (**47**–**53**).

**Figure 7:**
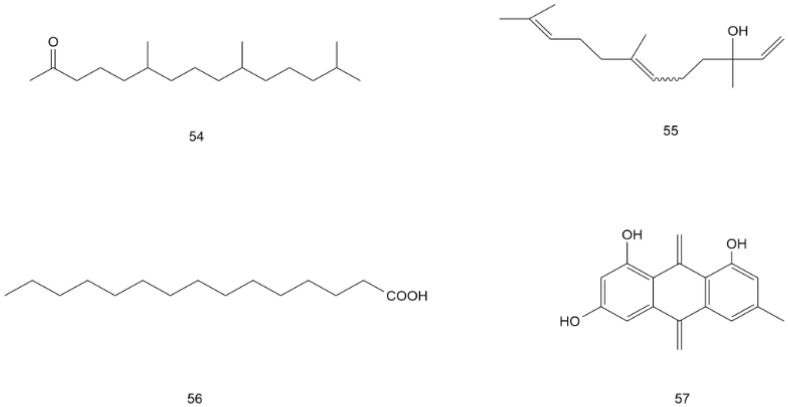
Structural formula of volatile components and other compounds in AG (**54**–**57**).

AG is rich in flavonoids, phenols, steroids, terpenoids, water-soluble polysaccharides, and other volatile components, including dihydromyricetin, myricetin, grossedentatasin, grossedentataside, quercetin, rutinum, quercetin-3-O-β-D-glucoside, gallic acid, gallicin, ethyl gallate, and gallic-β-D-glucoside [27,28,29,30], of which flavonoids are the most abundant compounds (up to 43%) [31], while dihydromyricetin (up to 30%, m/m) is the most abundant among the flavonoids [32,33]. This section summarizes the distribution, location, and molecular and structural formulae of the various compounds in AG by referring to other literature [34] (Table 2 and Figure 1, Figure 2, Figure 3, Figure 4, Figure 5, Figure 6 and Figure 7).

### 3.1. Flavonoids

Approximately 30 flavonoids have been isolated from AG, including dihydromyricetin, quercetin, myricetin, hesperidin, apigenin, and ampelopsin, among which 3-dihydroxyquercetin and dihydromyricetin are isomers of each other. Among the components, dihydromyricetin has the highest content and is the main active ingredient. The total flavonoid content of different leaf types also varies, with the highest content of 31.66% in medium-sized leaves and as high as 43.4% to 45.52% in tender stems and leaves.

Zhou [39], Zhou [50], and Liu et al. [51] were the first to isolate dihydromyricetin from a mixture of young stems and leaves of AG, which is only found in Ampelopsis of the grape family and is a chemical taxonomic characteristic of this genus [17]. Yuan et al. [43] first isolated two compounds, myricetin and myricetrines, from the aerial part of AG, and the contents of total flavonoids, dihydromyricetin, and myricetin in AG were 43.4–44.0%, 37.4–38.5%, and 1.72–1.78%, respectively. Qin et al. [52] extracted and isolated two flavonoid components, myricetin and dihydromyricetin, from the fine powder of AG of Yao nationality in Guangxi, and myricetin was isolated from this plant for the first time.

### 3.2. Phenols

Phenols are a pharmacodynamic component of AG. Phenolic compounds have been isolated, including gallic acid, gallicin, ethyl gallate, gallic-β-D-glucose, catechin, epicatechin, and epigallocatechin. Bai et al. [40] used column chromatography to isolate and identify eight phenols in AG samples in Zhangjiajie. These include dihydromyricetin (I), 5,7,3′,4′,5′-pentahydroxydihydroflavone (II), gallic β-D-glucoside (III), gallic acid (IV), ethyl gallate (V), myricetrin (VI), (2R, 3S)-5,7,3′,4′,5′-pentahydroxydihydroflavonol (VII), and myricetin (VIII). Wang et al. [45] first isolated and identified gallic acid using chromatography and spectral analysis. Wang [27] first isolated gallicin from the ethyl acetate extract of AG. Zhang [30] isolated gallic β-D-glucose from the mixture of spring and summer tender stems and leaves of AG.

### 3.3. Steroids and Terpenoids

Three steroidal compounds and one terpenoid have been isolated from AG, including stigmasterol, β-sitosterol, oleanolic acid, and the terpenoid ambrein [34]. Yuan et al. [43] first isolated ambrein (terpenoids) and β-sitosterol (steroids) from the aboveground part of AG. Wang et al. [45] first isolated stigmasterol using chromatography and spectral analysis. He et al. [36] isolated oleanolic acid using repeated silica gel, polyamide column chromatography, and recrystallization.

### 3.4. Water-Soluble Polysaccharide

Pan [53] extracted selenium-rich polysaccharide from fermented AG in Zhangjiajie Bachong Village using hot water extraction and ethanol precipitation. Under optimal extraction conditions, the extraction ratio of polysaccharides was 11.26%. Furthermore, the polysaccharide was composed of Man, GlcUA, Glc, Gal, and Xyl, which had the excellent scavenging ability of the 2,2-biphenyl-1-picrylhydrazyl (DPPH) free radical. Four proteins, AGP-3, AGP-4, ALPS, and ASPS, were further isolated from the water-soluble polysaccharides [54,55]. Zou et al. [56] used the hot water extraction method to extract the polysaccharide of AG. The results showed that in August, the polysaccharide content of the stems and leaves of AG in Huaihua City, Hunan Province, was the highest at 1.41% and 2.79%, respectively.

### 3.5. Volatile Components and Other Compounds

Three major volatile oil components, phytol, n-Hexadecanoic acid, and cedrol, were isolated from AG samples (Provided by the Science and Technology Commission of Yongding District, Zhangjiajie, China) using gas chromatography-mass spectrometry (GC-MS) analysis [46]. Zhang et al. [47] isolated and identified 63 volatile oil components of AG using solid phase microextraction/GC-MS, including ethanol, 1,3-di-tert-butyl benzene, 2-methyl decane, 2,4-dimethyl-1-decene, 2,4-dimethyl-1-heptene, 7-methy-11-undecene, 2,6-dimethyl-nonane, sabinene, α-pinene, ethylformate, tridecane, β-thujone, 1-undecene, 2,3,5,8-tetramethyl-decane, and 4.6-dimethyl-dodecane.

## 4. Pharmacological Properties

### 4.1. Anti-Inflammatory and Analgesia

Dihydromyricetin (purified moecule), the main active ingredient in AG, has a good anti-inflammatory and analgesic effect. Intraperitoneal injection of dihydromyricetin (purified moecule) into collagen-induced arthritis (CIA) mice showed a significant reduction in erythema and swelling of the paws after dihydromyricetin treatment, and the results of the pathological analysis of the knee joint and peripheral blood cytokine assay confirmed the anti-arthritic effect of dihydromyricetin [57]. Wu et al. [58] further demonstrated that dihydromyricetin (purified moecule) is protective against CIA in mice by blocking the phosphorylation of I-κB kinase and impeding the activation of nuclear factor-κB (NF-κB) to inhibit the formation of osteoclasts. Jia et al. [59] reported that dihydromyricetin (purified moecule) effectively inhibited rainfarin-induced production and expression of several pro-inflammatory cytokines (IL-1β, TNF-α, and IL-17) in mouse bone marrow-derived macrophages, thereby attenuating the pancreatic and systemic inflammatory responses in mice with acute pancreatitis (AP) induced by rainfarin injection. In a model of foot and plantar swelling and acute inflammation in rats caused by carrageenan gum, dihydromyricetin (purified moecule) significantly reduced carrageenan-induced paw foot swelling in rats, and the effect was similar to the anti-inflammatory effect of the positive control drugs indomethacin, dexamethasone, and methotrexate [60]. Cheng et al. observed that AG (crude plant extract) protects against ulcerative colitis (UC) in mice by inhibiting IRAK1/TAF6/ NF-κB-mediated inflammatory signaling pathways [61].

### 4.2. Anti-Oxidation

Various anti-oxidation experiments have shown that the antioxidant activity of flavonoid-rich AG extract is similar to that of tert-butylhydroquinone [62] and has high scavenging activity against 1,1-Diphenyl-2-picrylhydrazyl (DPPH) radicals [63]. AG extract and dihydromyricetin (purified moecule) exhibited strong DPPH radical scavenging capacity and high oxygen radical absorbance capacity during in vitro experiments, and this antioxidant activity may act through the activation of the cellular Nrf2/Keap 1 pathway [64]. The antioxidant effect of various AG components differs, among which the strongest scavenging ability for DPPH free radicals was observed in Myricetin (purified moecule), the strongest scavenging ability for O^2−^ was observed in dihydromyricetin, and the scavenging effect for hydroxyl radicals (OH-) was in the order myricetin > dihydromyricetin > total flavonoids of AG [65], and dihydromyricetin also had strong antioxidant activity in soybean oil, cooked beef, and sausage [66,67]. After constructing a hyperlipidemia (HLP) model in rats, AG flavonoid suspension was instilled, and the results showed that AG reduced serum total cholesterol, triglyceride, and low-density lipoprotein levels, increased high-density lipoprotein levels, reduced lipid peroxidation [68], improved the body’s antioxidant capacity, and improved blood lipid levels in HLP rats.

### 4.3. Reduction of Blood Sugar, Blood Pressure, and Blood Lipid Levels

Some studies have demonstrated the therapeutic efficacy of AG (crude plant extract) in preventing and treating triple highs (blood glucose, blood pressure, and blood lipids) in patients with adult type 2 diabetes mellitus (T2DM) who supplemented their daily diet with AG. The results showed a significant decrease in fasting plasma glucose, glycated albumin, cystatin C, and retinol-binding protein-4 levels in participants supplemented with AG, indicating that AG supplementation improves glycemic and renal function parameters in patients with adult T2DM and has a good hypoglycemic effect [69]. Furthermore, AG (crude plant extract) ameliorates glucose and lipid metabolism disorders in type 2 diabetic rats by inhibiting gluconeogenesis through the Akt/Foxo1/Pck2 signaling pathway and fatty acid synthesis through the SREBP1c/Fasn signaling pathway [70]. A hypertensive rat model was artificially created by surgery, followed by continuous gavage of an aqueous solution of AG (crude plant extract) and feeding for 30 days. Finally, blood pressure and heart rate values were detected, and the results showed that AG effectively lowered blood pressure but did not affect heart rate [71].

### 4.4. Liver and Kidney Protection

The treatment of hamsters on a high-fat diet with AG (crude plant extract) and dihydropyrimethamine (purified moecule) reduced high-fat-induced weight gain and liver lipid deposition and lowered serum TG and TC levels, indicating that AG and dihydropyrimethamine have a protective effect on hamster liver during a high-fat diet [72]. Three animal models of hyperuricemia induced by yeast, hypoxanthine, and potassium oxyzincate were constructed and treated with AG total flavonoids by gavage after successful modeling. Compared with the control group, the serum uric acid (UA) content, xanthine oxidase (XOD), and adenosine deaminase (ADA) activities of mice in the AG total flavonoids group were significantly reduced. Total flavonoids of AG exerted anti-hyperuricemia effects by reducing serum UA levels, inhibiting XOD and ADA enzyme activities, and improving UA metabolism, with certain effects on improving kidney injury and preserving kidney function [73]. Wu et al. [74] established a mouse model of hyperuricemia by intraperitoneal injection of potassium oxyzincate and gavaged AG extract for 14 days. The serum UA, creatinine, urea nitrogen, aspartate amino transaminase, glutamate amino transaminase, and liver XOD levels of mice in the AG extract group showed a decreasing trend. The pathological histomorphological examination showed that AG significantly improved the liver and kidney tissues of mice with hyperuricemia, indicating that it effectively lowered UA levels and protected the liver and kidney in mice with hyperuricemia. Human experiments have also shown that AG extract reduces postprandial UA levels by inhibiting XOD activity and promoting the excretion of UA [75].

### 4.5. Tumor Suppression and Anti-Tumor Activity

Studies have demonstrated that dihydromyricetin (purified moecule) has anti-cancer, anti-tumor, and atherosclerotic properties [76]. Xylene was used to induce acute inflammation in the auricles of mice, followed by the administration of different doses of AG total flavonoids. The swelling of the auricles was inhibited in all groups of mice treated with AG total flavonoids, and the higher the concentration of AG total flavonoids, the higher the inhibition rate of swelling in the auricles of mice [77]. Zhang et al. reported that dihydromyricetin (purified moecule) inhibited the migration and invasion of SK-Hep-1 and MHCC97L hepatocellular carcinoma cell lines by downregulating the expression of MMP-9 protein [78]. The compound dihydromyricetin isolated from AG healing tissues exhibited desirable cytotoxic activity against mouse breast cancer (4T1), human lung adenocarcinoma (A549), and human non-small cell lung cancer (NCI-H1975) cell lines [79], providing a new direction for developing anti-cancer drugs. Dihydromyricetin (purified moecule) also inhibits the growth of cholangiocarcinoma cell lines by increasing the expression of miR-455-3p, which ultimately inhibits tumor growth [80].

Dihydromyricetin (purified moecule) inhibits the expression of nasopharyngeal carcinoma cell lines by blocking the Wnt/β-linked protein signaling pathway and regulating the expression of downstream proteins in nasopharyngeal carcinoma [81], thus serving as a novel agent for treating nasopharyngeal carcinoma. In addition, it downregulates the expression of cyclins A1, D1, SMAD3, and SMAD4 to inhibit cell cycle arrest and ultimately inhibit the proliferation of human choriocarcinoma cells [82]. In contrast, the inhibition of ovarian cancer (OC) cells is mainly due to the upregulation of cleaved cystatin-3 and Bax/Bcl-2 in OC cells, which ultimately induces apoptosis [83]. Dihydromyricetin can also be combined with multiple drugs (adriamycin, nedaplatin, oxaliplatin, nedaplatin, erlotinib, and paclitaxel) to increase the sensitivity of cancer cells to drugs, reverse multidrug resistance, and exert synergistic anti-cancer effects [84].

### 4.6. Antibacterial

AG extract and its active ingredient, dihydromyricetin (purified moecule), showed good antibacterial activity against *Staphylococcus aureus* [85]. Xiao-Nian Xiao evaluated the antibacterial ability of dihydromyricetin (purified moecule) extracted from AG using the disc diffusion method [86] and showed that dihydromyricetin exhibited desirable antibacterial activity against five food-borne bacteria (*Staphylococcus aureus, Bacillus subtilis, Escherichia coli, Salmonella paratyphi,* and *Pseudomonas aeruginosa*), causing lysis of bacterial cell walls, leakage of intracellular components, and inhibition of the bacterial tricarboxylic acid cycle pathway, ultimately leading to bacterial death. In vitro antibacterial tests with AG powder and dihydromyricetin (purified moecule) against *Aeromonas vivax* and *Aeromonas hydrophila* showed good antibacterial effects [87]. Muhammad Umair et al. [88] used the agar pore diffusion method to evaluate the antibacterial activity of AG extracts and isolated six compounds from AG extracts. Approximately 5,7,8,3,4-pentahydroxyisoflavone (C_15_H_10_O_7_) exhibited the strongest antibacterial activity against *Bacillus cereus* (AS11846) and *Staphylococcus aureus* (CMCCB26003) and can be used as a potent antibacterial agent in the food processing industry.

## 5. Toxicology

Many trials have verified the safety of the clinical use of AG. Zhong et al. [89] administered the total flavonoids of Guangxi AG to rats at doses of 1.5 g/kg and 0.3 g/kg for 12 weeks. Two weeks after the Guangxi AG was discontinued, the appearance, behavior, body weight, organ coefficient, and blood biochemistry indices of the rats were not significantly different from those of the control group. Furthermore, no obvious lesions related to drug toxicity were observed in the pathological examination, and there was no delayed toxic reaction after discontinuing the drug, indicating that Guangxi AG flavonoid had no significant toxic effect on rats after long-term administration. In another study [90], three gavages (0.2 mL/10 g/bodyweight) were administered three hours apart to mice that were observed for two weeks. Three mutagenicity tests were negative. The mice had good growth and development, with no adverse effects on body weight or food utilization. Furthermore, the routine blood and white blood cell levels of the dose group were not significantly different from those of the control group. Furthermore, no substantial pathological changes were observed in the organs, indicating that the Enshi selenium-rich AG used in the test was safe and non-toxic.

## 6. Conclusions

This review summarizes the various uses of AG and its morphology, phytochemistry, pharmacology, and toxicology. Furthermore, we summarized the use of AG in different regions and the molecular and structural formulas of various components based on previous studies. AG is mainly used to treat jaundice-type hepatitis, cold, and fever and lower blood lipid and sugar levels. Moreover, single compounds of AG, such as flavonoids and dihydromyricetin, have various biological activities, including anti-inflammatory, analgesic, anti-tumor, and antibacterial properties.

Dihydromyricetin significantly inhibits certain cancer cells, suggesting that dihydromyricetin, a component of AG, may serve as a novel anti-cancer drug or as a synergistic drug with existing anti-cancer drugs, providing a new direction for anti-cancer and anti-tumor therapy.

## Figures and Tables

**Table 1:** Effects of rattan tea in different regions.

Nationality/Region	Nickname	Site of Use	Role of Tradition
Fujian Hakka	Ampelopsis grossedentata	Stem and leaf	Clearing heat and moisturizing the lung, anti-inflammation and detoxification, reducing blood pressure and fat, and eliminating fatigue [10].
SHE-Minority in Sanming, Fujian Province	AG	Young stems and leaves	Heat stroke, mouth sores, aphonia, toothache, equine dental sores, and foot eczema [11].
Guangxi (Zhuang nationality)	Sweet tea	Leaves and shoots	Good medicine for clearing heat and moisturizing the lung, eliminating phlegm and cough, and stopping bleeding and swelling [12].
Hubei (Tujia Family)	Musty Tea	Leaves and shoots	Drinking tea can prevent and treat hypertension, and external application of fresh plants can treat Carbuncle swelling [13].
Xiangxi (Hmong)	AG	Young leaf	Cool and quench thirst, as one of the teas for “oil tea” [14].
Yao nationality	Tian Po tea, AG	The whole plant was used as medicine	Treatment of throat swelling and pain, cold and fever, icteric hepatitis, sore boils, anti-inflammatory, antibacterial, reducing three high, liver protection, liver protection, antioxidant, anti-tumor [15,16].
Fanjing Mountain area, Guizhou province	AG, Sweet tea, White tea, Bang Bang tea	Tender stem and leaves	Prevention and treatment of hypertension, treatdamp-heat dysentery, pruritus of the skin, and ulcer or ulcer. It has the functions of nourishing the liver and kidney, moistening the lungs, relieving coughs, relieving drowsiness, and promoting sobriety [6].
JiNuo nationality	AG	^★^	Chewing and swallowing to treat toothache [17].
Dong nationality	AG	The whole plant was used as medicine	Beverage tea and treat skin and external diseases [18].
LaHu nationality	AG	^★^	Daily consumption of tea [18].
Hengdong County, Hunan Province	AG	^★^	People used to treat cuts, falls, swollen gums, oral ulcers, gastric ulcers, influenza, pneumonia, hypertension, diabetes, osteoporosis, hemorrhoids, constipation, anti-drinking poisoning, and cardiovascular diseases [19].
Yingde city, Lianzhou city, Guangdong province	Wild AG, AG, White tea, Lai Li tea, Nectar tea	^★^	Treat colds and fevers, sore throats, icteric hepatitis, sore boils, hypertension, hyperlipidemia, etc. [20].

Note: ^★^ It shows that there is no detailed record of the use site of AG in this area.

**Table 2:** Various chemical compositions ^1^ in AG.

Active Component		Molecular Formula	Distribution	References
Flavonoids				
**1**	Myricetin	C_15_H_10_O_8_	tender stem and leaf	[35]
**2**	Kaempferol	C_15_H_10_O_6_	stem and leaf	[36]
**3**	Quercetin	C_15_H_10_O_7_	tender stem and leaf	[37]
**4**	Myricetin-3′-O-β-D-xylopyranoside	C_15_H_9_O_7_	leaf	[38]
**5**	Dihydrokaempferol	C_15_H_12_O_6_	leaf	[38]
**6**	Dihydroquercetin	C_15_H_12_O_7_	stem and leaf	[36]
**7**	Dihydromyricetin	C_15_H_12_O_8_	tender stem and leaf	[39]
**8**	Taxifolin	C_15_H_12_O_7_	stem and leaf	[27]
**9**	(2R,3S)-5,7,3′,4′,5′-pentahydroxyflavanonol	C_15_H_12_O_8_	stem and leaf	[40]
**10**	6,7-dihydroxy-3′-methoxy-4′,5′-methylenedioxyisoflavone	C_17_H_12_O_7_	stem and leaf	[41]
**11**	6,7-dihydroxy-3′-methoxy-4, 5′-methylenedioxyisoflavone 6-O-β-D-glucopyranoside	C_17_H_11_O_6_	stem and leaf	[41]
**12**	6,7-dihydroxy-3′-methoxy-4′,5′-methylenedioxyisoflavone 6-O-α-L-rhamnopyranoside	C_17_H_11_O_6_	stem and leaf	[41]
**13**	6,7-dihydroxy-3′-methoxy-4′,5′-methylenedioxyisoflavone 6-O-β-D-xylopyranosyl-(1-6)-β-D-glucopyranoside	C_17_H_11_O_6_	stem and leaf	[41]
**14**	Hesperetin	C_15_H_13_O_6_	stem and leaf	[36]
**15**	5,7,3′,4′,5′-pentahydroxyflavanone	C_15_H_12_O_7_	stem and leaf	[40]
**16**	Grossedentatasin	C_16_H_15_O_3_	stem and leaf	[28]
**17**	Grossedentataside	C_16_H_15_O_3_	stem and leaf	[28]
**18**	Luteolin	C_15_H_10_O_6_	stem	[42]
**19**	Vitexin	C_15_H_9_O_5_	stem	[42]
**20**	Myricetrin	C_15_H_9_O_7_	aerial part	[43]
**21**	Rutinum	C_15_H_9_O_6_	tender stem and leaf	[37]
**22**	Myricetin-3-O-β-D-galactopyranoside	C_15_H_9_O_7_	stem and leaf	[42]
**23**	Apigenin	C_15_H_10_O_5_	stem and leaf	[36]
**24**	5,7-dihydroxy-3′4′-dihydroxyflavone-3-O-6″-rhamnose	C_15_H_9_O_6_	leaf	[44]
**25**	5,7-dihydroxy-3′4′5′-trihydroxyflavone-3-O-6″-rhamnose	C_15_H_9_O_7_	leaf	[44]
**26**	Afzelechin	C_15_H_9_O_6_	stem and leaf	[27,38]
**27**	Astragalin	C_15_H_9_O_5_	stem and leaf	[27,38]
**28**	Quercetin-3-O-α-L-rhamnopyranoside	C_15_H_9_O_6_	stem and leaf	[27,38]
**29**	Quercetin-3-O-β-D-glucoside	C_15_H_9_O_6_	stem and leaf	[27,38]
**30**	Myricetin-3-O-β-D- galactoside	C_15_H_9_O_7_	stem and leaf	[27,38]
**31**	Bellidifolin	C_14_H_10_O_6_	stem and leaf	[43]
Phenols				
**32**	Gallic acid	C_7_H_6_O_5_	tender stem and leaf	[45]
**33**	Gallicin	C_8_H_8_O_5_	stem and leaf	[27]
**34**	Ethyl gallate	C_9_H_10_O_5_	tender stem and leaf	[30]
**35**	Gallic-β-D-glucose	C_7_H_5_O_5_	tender stem and leaf	[30]
Steroids and terpenoids				
**36**	Stigmasterol	C_29_H_48_O	tender stem and leaf	[45]
**37**	β-sitosterol	C_29_H_50_O	tender stem and leaf	[45]
**38**	Oleanolic acid	C_30_H_48_O_3_	stem and leaf	[36]
**39**	Ambrein	C_30_H_52_O	aerial part	[43]
Volatile components and other compounds				
**40**	Phytol	C_20_H_40_O	tender stem and leaf	[46]
**41**	n-Hexadecanoic acid	C_15_H_30_O_2_	tender stem and leaf	[46]
**42**	Cedrol	C_15_H_26_O	tender stem and leaf	[46]
**43**	β-thujone	C_10_H_16_O	stem and leaf	[47]
**44**	β-cyclocitral	C_10_H_16_O	stem and leaf	[48]
**45**	(E)-2-hexenal	C_6_H_10_O	stem and leaf	[49]
**46**	(Z)-3-hexenyl hexanote	C_4_H_6_O_2_	stem and leaf	[49]
**47**	Trimethyl pyrazine	C_7_H_10_N_2_	stem and leaf	[49]
**48**	Phenylacetaldehyde	C_8_H_8_O	stem and leaf	[49]
**49**	α-terpinol	C_10_H_20_O	stem and leaf	[49]
**50**	Methyl salicylate	C_8_H_8_O_3_	stem and leaf	[49]
**51**	Geraniol	C_10_H_18_O	stem and leaf	[49]
**52**	β-ionone	C_13_H_20_O	stem and leaf	[49]
**53**	(Z)-jasmone	C_10_H_14_O	stem and leaf	[49]
**54**	6,10,14-trimethyl-2-pen-tadecanone	C_18_H_36_O	stem and leaf	[49]
**55**	Nerolidol	C_15_H_26_O	stem and leaf	[49]
**56**	Palmitic acid	C_15_H_30_O_2_	stem and leaf	[49]
**57**	Emodin	C_17_H_14_O_3_	stem and leaf	[49]

^1^ Two-dimensional structural formula of various components was drawn using Kingdraw software, v5.0.

## Data Availability

Source databases for these publications include the Science Citation Index (SCI), SCI Expanded, and the Chinese Science Citation Database.
